# Fusion-Expressed CTB Improves Both Systemic and Mucosal T-Cell Responses Elicited by an Intranasal DNA Priming/Intramuscular Recombinant Vaccinia Boosting Regimen

**DOI:** 10.1155/2014/308732

**Published:** 2014-04-01

**Authors:** Sugan Qiu, Xiaonan Ren, Yinyin Ben, Yanqin Ren, Jing Wang, Xiaoyan Zhang, Yanmin Wan, Jianqing Xu

**Affiliations:** ^1^Shanghai Public Health Clinical Center, Fudan University, Shanghai 201508, China; ^2^Institute of Biomedical Science, Fudan University, Shanghai 200032, China

## Abstract

Previous study showed that CTB (Cholera toxin subunit B) can be used as a genetic adjuvant to enhance the systemic immune responses. To further investigate whether it can also be used as a genetic adjuvant to improve mucosal immune responses, we constructed DNA and recombinant Tiantan vaccinia (rTTV) vaccines expressing OVA-CTB fusion antigen. Female C57BL/6 mice were immunized with an intranasal DNA priming/intramuscular rTTV boosting regimen. OVA specific T-cell responses were measured by IFN-**γ** ELISPOT and specific antibody responses were determined by ELISA. Compared to the nonadjuvant group (pSV-OVA intranasal priming/rTTV-OVA intramuscular boosting), pSV-OVA-CTB intranasal priming/rTTV-OVA-CTB intramuscular boosting group significantly improved the magnitudes of T-cell responses at spleen (1562 ± 567 SFCs/10^6^ splenocytes versus 330 ± 182 SFCs/10^6^ splenocytes, *P* < 0.01), mesenteric LN (96 ± 83 SFCs/10^6^ lymphocytes versus 1 ± 2 SFCs/10^6^ lymphocytes, *P* < 0.05), draining LNs of respiratory tract (109 ± 60 SFCs/10^6^ lymphocytes versus 2 ± 2 SFCs/10^6^ lymphocytes, *P* < 0.01) and female genital tract (89 ± 48 SFCs/10^6^ lymphocytes versus 23 ± 21 SFCs/10^6^ lymphocytes, *P* < 0.01). These results collectively demonstrated that fusion-expressed CTB could act as a potent adjuvant to improve both systemic and mucosal T-cell responses.

## 1. Introduction

DNA vaccines are insufficient to stimulate strong mucosal and systemic immunity when inoculated alone [1]. Various approaches have been taken to improve the immunogenicity of DNA vaccine, such as delivering DNA by using electroporation or enhancing host response by coadminstration of genetic adjuvants [1].

Cholera toxin (CT) is a strong mucosal immunogen as well as an effective adjuvant [2]; both the holotoxin and its subunits can be used as adjuvants for protein based vaccines [3, 4]. Recent studies suggested that both CTA (Cholera toxin subunit A) and CTB (Cholera toxin subunit B) can also be used as genetic adjuvants to boost the systemic immune responses elicited by DNA vaccines [5, 6]. To investigate whether CTB can also be used as a genetic adjuvant to improve antigen specific mucosal immune responses, in this study, we constructed DNA and recombinant Tiantan vaccinia (rTTV) vaccines encoding OVA-CTB fusion antigen and tested their immunogenicity in an intranasal DNA priming/intramuscular rTTV boosting regimen, which has been proved to be able to raise vigorous mucosal and systemic immune response [7].

## 2. Materials and Methods

### 2.1. Vaccines and Mice

All DNA and recombinant vaccinia virus vaccines were constructed in our previous work. The 6–8-week-old female C57BL/6 mice were bred and maintained under specific pathogen-free condition. All animal experiments were reviewed by the Institutional Animal Care and Use Committee (IACUC) of Shanghai Public Health Clinical Center.

### 2.2. Mice Immunization and Sampling

DNA vaccine (5 *μ*g/mouse) mixed with Turbofect (Fermentas, Cat. number R0541) was given intranasally on weeks 0, 2, and 4. And on week 7, mice were boosted intramuscularly with recombinant Tiantan vaccinia vaccine (1 × 10^6^ pfu/mouse). Two weeks after the final vaccination, mice were euthanized. Vaginal lavage, bronchial alveolar lavage, and serum were collected for the detection of specific antibody response. Spleen, cervical, axillary, iliac, inguinal, and mesenteric lymph nodes were isolated for T-cell response assay.

### 2.3. IFN-*γ* ELISPOT Assay

Freshly isolated mouse splenocytes were adjusted to the concentration of 4 × 10^6^ cells/mL and plated into 96-well ELISPOT plate (BD Bioscience, Cat. number 551083) coated with anti-mouse IFN-*γ* antibody at 50 *μ*L/well (2 × 10^5^ cells/well). The splenocytes were stimulated with OVA peptide (amino acids 257–264) at the final concentration of 5 *μ*g/mL. After incubation at 37°C with 5% CO_2_ for 20 hours, the ELISPOT plates were developed according to the manufacturer's manual and read with Immunospot Reader (ChampspotIII, Beijing Sage Creation Science, China).

### 2.4. ELISA Assay

ELISA plates coated with 2 *μ*g/mL OVA were used for the detection of anti-OVA antibodies (Abs). Serum, bronchial lavage, or vaginal lavage samples were 2-fold serially diluted in PBS containing 5% skimmed milk and 0.5% TWEEN-20. OVA specific IgG and IgA were detected by peroxidase conjugated anti-mouse IgG and anti-mouse IgA, respectively. End point titers were determined by the last dilution, whose OD was beyond or equal to 2-fold that of the corresponding dilution of mice sera immunized with mock control.

### 2.5. Statistical Analysis

Comparisons between two groups were done by the method of unpaired *t*-test and comparisons among three or more groups were done by using the method of one-way ANOVA (Prism6, GraphPad Software, Inc.). Significant difference was defined as *P* ≤ 0.05.

## 3. Results

### 3.1. Systemic Immune Responses

Mice were immunized according to the schedule shown in Table 1. Two weeks after the final immunization, splenocytes were isolated and OVA-specific T-cell responses were quantified by IFN-*γ* ELISPOT assay. Specific binding antibody in serum was detected by ELISA.

**Figure 1:**
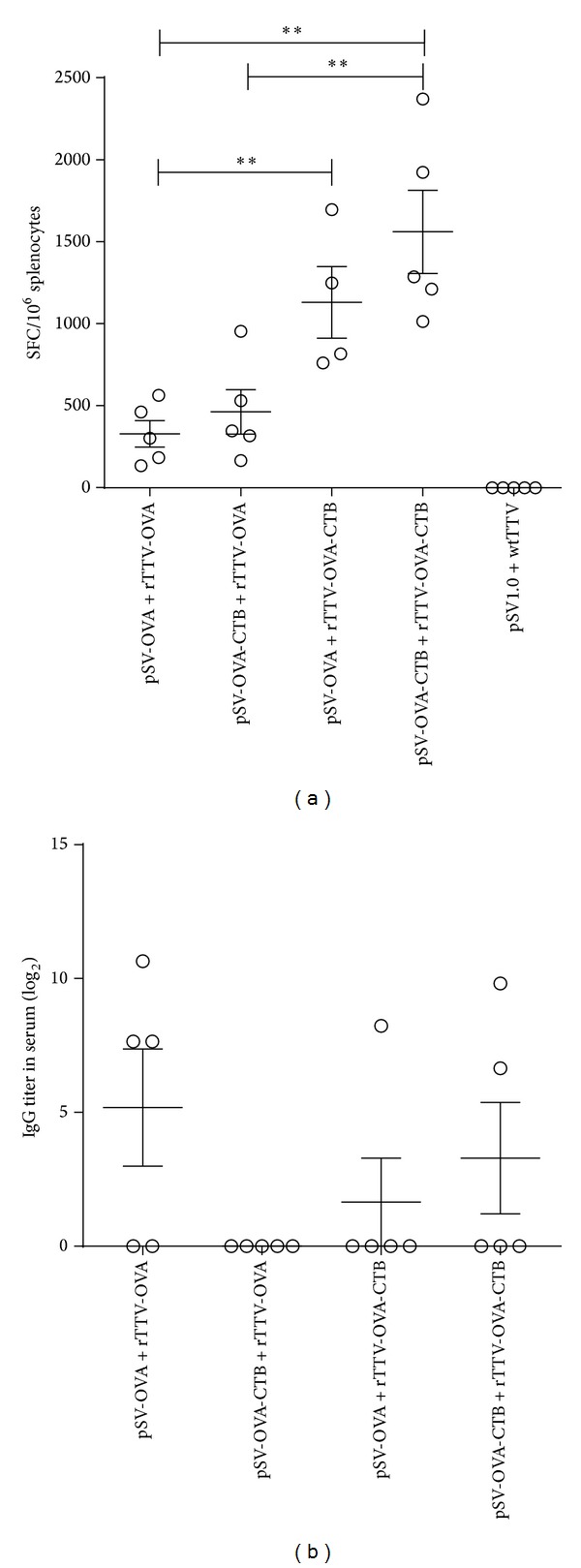
Humoral and cellular immune responses at systemic level. (a) Ovalbumin specific T-cell responses in spleen. The cellular responses elicited in rTTV-OVA-CTB boosting groups were significantly stronger than those elicited in rTTV-OVA boosting groups. (b) Ovalbumin specific antibody responses in serum. OVA specific IgG titers elicited by adjuvant groups tended to be lower than the nonadjuvant group, but no statistical significance was reached. **P < 0.01.

ELISPOT results showed that all the rTTV-OVA-CTB boosting groups mounted significantly stronger T-cell immune responses (1132 ± 436 SFCs/10^6^ splenocytes for pSV-OVA intranasal priming/rTTV-OVA-CTB intramuscular boosting group and 1562 ± 567 SFCs/10^6^ splenocytes for pSV-OVA-CTB intranasal priming/rTTV-OVA-CTB intramuscular boosting group) than rTTV-OVA boosting groups (330 ± 182 SFCs/10^6^ splenocytes for pSV-OVA intranasal priming/rTTV-OVA intramuscular boosting group and 464 ± 303 SFCs/10^6^ splenocytes for pSV-OVA-CTB intranasal priming/rTTV-OVA intramuscular boosting group) (Figure 1(a)). OVA specific IgG titers elicited by adjuvant groups tended to be lower than the nonadjuvant group, but no statistical significance was observed (Figure 1(b)).

### 3.2. Humoral and Cellular Immune Responses Elicited in Respiratory Tract

**Figure 2:**
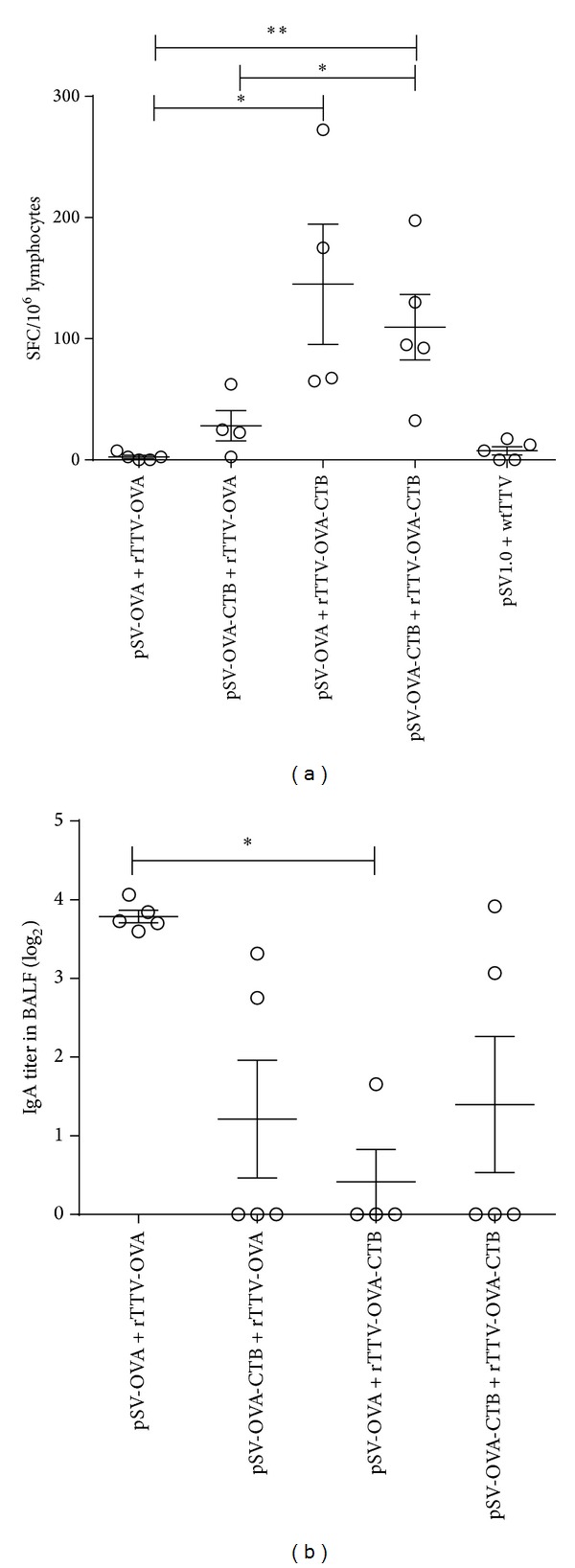
Specific antibody and T-cell immune responses elicited in respiratory tract. (a) Ovalbumin specific T-cell response in cervical and axillary lymph nodes. Significant differences were observed between rTTV-OVA-CTB boosting groups and rTTV-OVA boosting groups. (b) Specific IgA titer in bronchial alveolar lavage. The average OVA specific IgA titer induced by pSV-OVA priming/rTTV-OVA boosting was significantly higher than pSV-OVA priming/rTTV-OVA-CTB boosting group. *P < 0.05, **P < 0.01.

We collected the bronchi alveolar lavage for specific IgA titration, cervical, and axillary lymph nodes for analysis of mucosal T-cell responses. The ELISPOT data showed that pSV-OVA intranasal priming/rTTV-OVA-CTB intramuscular boosting induced the highest T-cell responses (145 ± 99 SFCs/10^6^ lymphocytes) and pSV-OVA-CTB intranasal priming/rTTV-OVA-CTB intramuscular boosting group was the second (109 ± 60 SFCs/10^6^ lymphocytes). Both were significantly higher than the nonadjuvant group (pSV-OVA intranasal priming/rTTV-OVA intramuscular boosting, 2 ± 2 SFCs/10^6^ lymphocytes) (Figure 2(a)).

The mean titer of OVA specific IgA in bronchi alveolar lavage induced by adjuvant groups was lower than the nonadjuvant group. Significant difference was observed between pSV-OVA intranasal priming/rTTV-OVA intramuscular boosting group and pSV-OVA intranasal priming/rTTV-OVA-CTB intramuscular boosting group (Figure 2(b)).

### 3.3. Humoral and Cellular Immune Responses Elicited in Female Genital Tract

**Figure 3:**
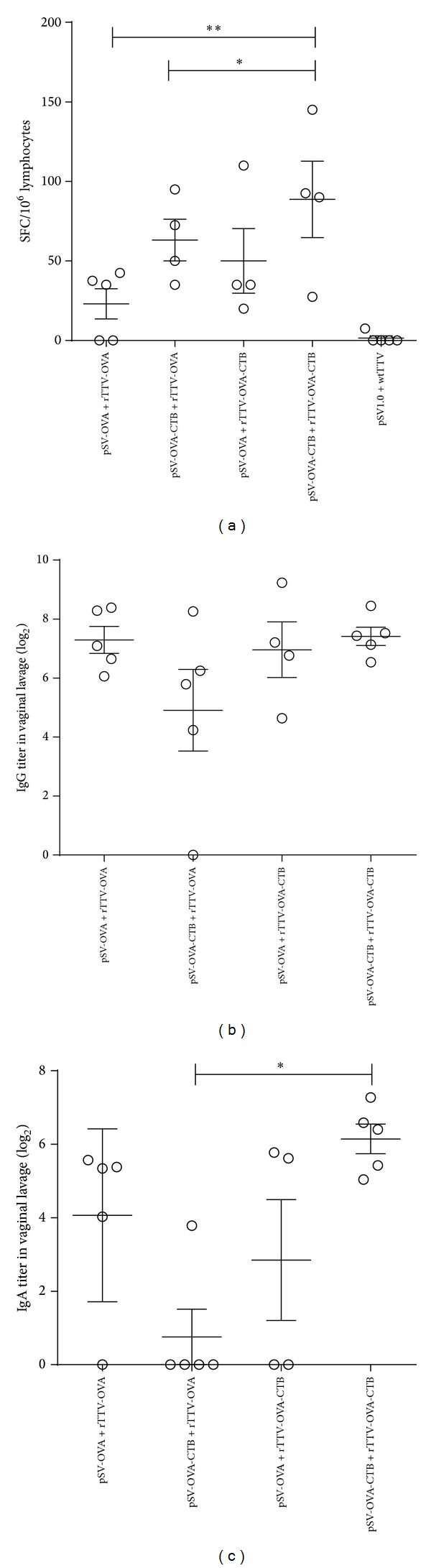
Specific antibody and T-cell immune responses elicited in female genital tract. (a) T lymphocytes responses in inguinal and iliac lymph nodes. The T-cell responses of rTTV-OVA-CTB boosting groups were significantly higher than those of rTTV-OVA boosting groups. (b) Specific IgG responses in vaginal lavage. No significant difference was found among different groups. (c) Specific IgA responses in vaginal lavage. IgA titer elicited by pSV-OVA-CTB priming/rTTV-OVA-CTB boosting group was significantly higher than pSV-OVA priming/rTTV-OVA-CTB boosting group. *P < 0.05, **P < 0.01.

Inguinal and iliac LNs were collected for ELISPOT assay and vaginal lavage was collected for specific IgA and IgG titration. T-cell responses of pSV-OVA-CTB intranasal priming/rTTV-OVA-CTB intramuscular boosting group (89 ± 48 SFCs/10^6^ lymphocytes) were significantly higher than rTTV-OVA boosting groups (23 ± 21 SFCs/10^6^ lymphocytes for pSV-OVA intranasal priming/rTTV-OVA intramuscular boosting group; 63 ± 26 SFCs/10^6^ lymphocytes for pSV-OVA-CTB intranasal priming/rTTV-OVA intramuscular boosting group) (Figure 3(a)).

No significant difference was observed among the OVA specific IgG titers in vaginal lavage of all groups (Figure 3(b)). OVA specific IgA titers of pSV-OVA-CTB intranasal priming/rTTV-OVA-CTB intramuscular boosting group were significantly higher than pSV-OVA intranasal priming/rTTV-OVA-CTB intramuscular boosting group (Figure 3(c)).

### 3.4. Ovalbumin Specific T-Cell Responses in Mesenteric Lymph Nodes

**Figure 4:**
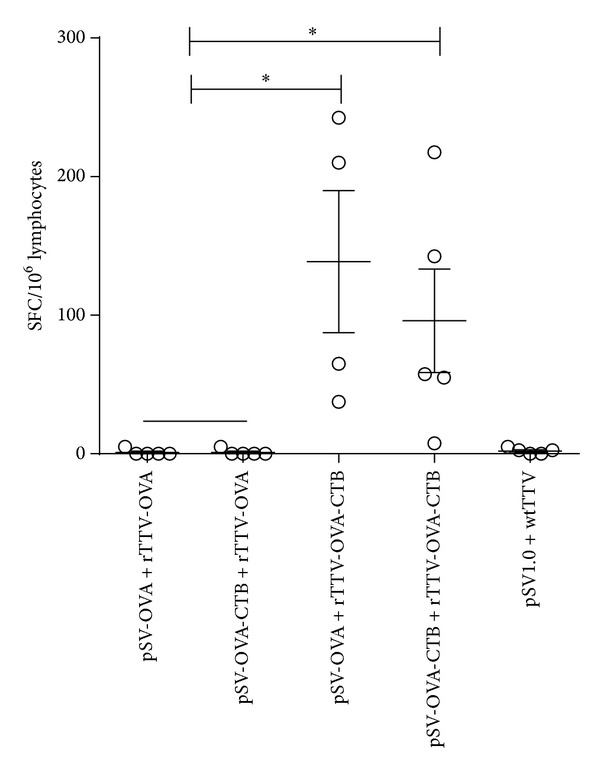
T-cell responses elicited in mesenteric lymph node. rTTV-OVA-CTB boosting raised significantly more rigorous T-cell responses than rTTV-OVA boosting groups. *P < 0.05.

To investigate specific T-cell responses in intestinal mucosa, we isolated mesenteric LN and measured OVA specific T-cell responses by ELISPOT assay. The results suggested that both rTTV-OVA-CTB boosting groups (138 ± 102 SFCs/10^6^ lymphocytes for pSV-OVA intranasal priming/rTTV-OVA-CTB intramuscular boosting group and 96 ± 84 SFCs/10^6^ lymphocytes for pSV-OVA-CTB intranasal priming/rTTV-OVA-CTB intramuscular boosting group) elicited significantly higher T-cell responses than rTTV-OVA boosting groups (1 ± 2 SFCs/10^6^ lymphocytes for pSV-OVA intranasal priming/rTTV-OVA intramuscular boosting group and 1 ± 2 SFCs/10^6^ lymphocytes for pSV-OVA-CTB intranasal priming/rTTV-OVA intramuscular boosting group) (Figure 4).

## 4. Discussion 

The mucosae constitute the major portal of entry of infectious agents. An ideal vaccine should induce both systemic and mucosal immune responses in order to block the entry of pathogens and contain infections* in vivo*. Majority of previous studies seek to induce mucosal immune responses by mucosal immunization [8–11]; however, this may not be applicable for naked DNA vaccines due to the relative weak immunogenicity [7].

In this study, we constructed DNA and recombinant Tiantan vaccinia (rTTV) vaccines encoding OVA-CTB fusion antigen and immunized C57/BL mice in an intranasal DNA priming/intramuscular rTTV boosting regimen. Our data showed that pSV-OVA-CTB priming (i.n.)/rTTV-OVA-CTB boosting (i.m.) elicited the highest magnitude of T-cell responses in spleen (system level). And the genetic adjuvant effect of CTB was more significant for recombinant vaccinia vaccine than for DNA vaccine, since the T-cell responses induced by pSV-OVA priming (i.n.)/rTTV-OVA-CTB boosting (i.m.) were significantly higher than pSV-OVA priming (i.n.)/rTTV-OVA boosting (i.m.) and the T-cell responses elicited by pSV-OVA-CTB priming (i.n.)/rTTV-OVA boosting (i.m.) were only slightly higher than the nonadjuvant group. In contrast, OVA specific IgG responses elicited by adjuvant groups tended to be lower than the nonadjuvant group although no statistical significance was reached.

We further tested specific immune responses at different mucosal sites and found that pSV-OVA-CTB priming (i.n.)/rTTV-OVA-CTB boosting (i.m.) consistently raised significant higher cellular immune response than the nonadjuvant group at respiratory, intestinal, and female genital tract, which indicated that the fused-expression of CTB in both DNA and rTTV vaccines is essential for eliciting robust T-cell responses at mucosal sites. Very interestingly, we found that rTTV-OVA-CTB boosting was especially efficient at improving specific T-cell responses in mesenteric lymph node. Besides, similar to the observations of antibody responses in serum, we found that the adjuvant groups tended to induce lower specific IgA titer in both bronchi alveolar lavage and vaginal lavage.

When being used as an adjuvant of protein vaccine, CTB can enhance specific immune response through either GM1 receptor-mediated antigen uptake [12] or stimulating expression of B7.2 on APCs [13]. As OVA is a secretory protein, we thus postulate that the secreted OVA-CTB fusion expressed by the DNA or recombinant vaccinia vaccines can also bind with GM1 receptor or interact with APCs* in vivo*, which may facilitate the uptake and presentation of OVA. This hypothesis was supported by our previous work, in which we found that the adjuvant effect decreased when separating CTB and the antigen into two plasmids (see Supplementary Figure  1 available online at http://dx.doi.org/10.1155/2014/308732). Further experiments will be conducted to confirm the proposed mechanisms and clarify the missing details.

## 5. Conclusions

Taken together, in spite of being short of mechanistic explanation, our data clearly showed that fusion-expressed CTB could serve as a potent adjuvant to enhance both systemic and mucosal T-cell response, not only for DNA vaccine but also for viral vectored vaccine.

## Supplementary Material

The fusion expressed CTB could improve specific T cell responses elicited by TRIVN (Tat, Rev, Integrase, Vif and Nef fusion antigen derived from HIV-1). However, when separating TRIVN and CTB into two DNA vaccines, the genetic adjuvant effect decreased.

## Figures and Tables

**Table 1:** Mice immunization schedule.

Group	No. of mice	Week 0 (50 *μ*g/mouse)	Week 2 (50 *μ*g/mouse)	Week 4 (50 *μ*g/mouse)	Week 7 (1E6pfu/mouse)	Week 9
A	5	pSV1.0	pSV1.0	pSV1.0	WT TTV	Euthanized
B	5	pSV-OVA	pSV-OVA	pSV-OVA	rTTV-OVA	Euthanized
C	5	pSV-OVA	pSV-OVA	pSV-OVA	rTTV-OVA-CTB	Euthanized
D	4	pSV-OVA-CTB	pSV-OVA-CTB	pSV-OVA-CTB	rTTV-OVA	Euthanized
E	5	pSV-OVA-CTB	pSV-OVA-CTB	pSV-OVA-CTB	rTTV-OVA-CTB	Euthanized
