# Supplementary material for: Fusion-Expressed CTB Improves Both Systemic and Mucosal T-Cell Responses Elicited by an Intranasal DNA Priming/Intramuscular Recombinant Vaccinia Boosting Regimen
Source: J Immunol Res. 2014 Apr 1;2014:308732. doi: 10.1155/2014/308732 (PMC3988707; doi:10.1155/2014/308732)
Supplement: Supplementary file 1 — The fusion expressed CTB could improve specific T cell responses elicited by TRIVN (Tat, Rev, Integrase, Vif and Nef fusion antigen derived from HIV-1). However, when separating TRIVN and CTB into two DNA vaccines, the genetic adjuvant effect decreased. [file 308732.f1.pptx]

## Slide 1
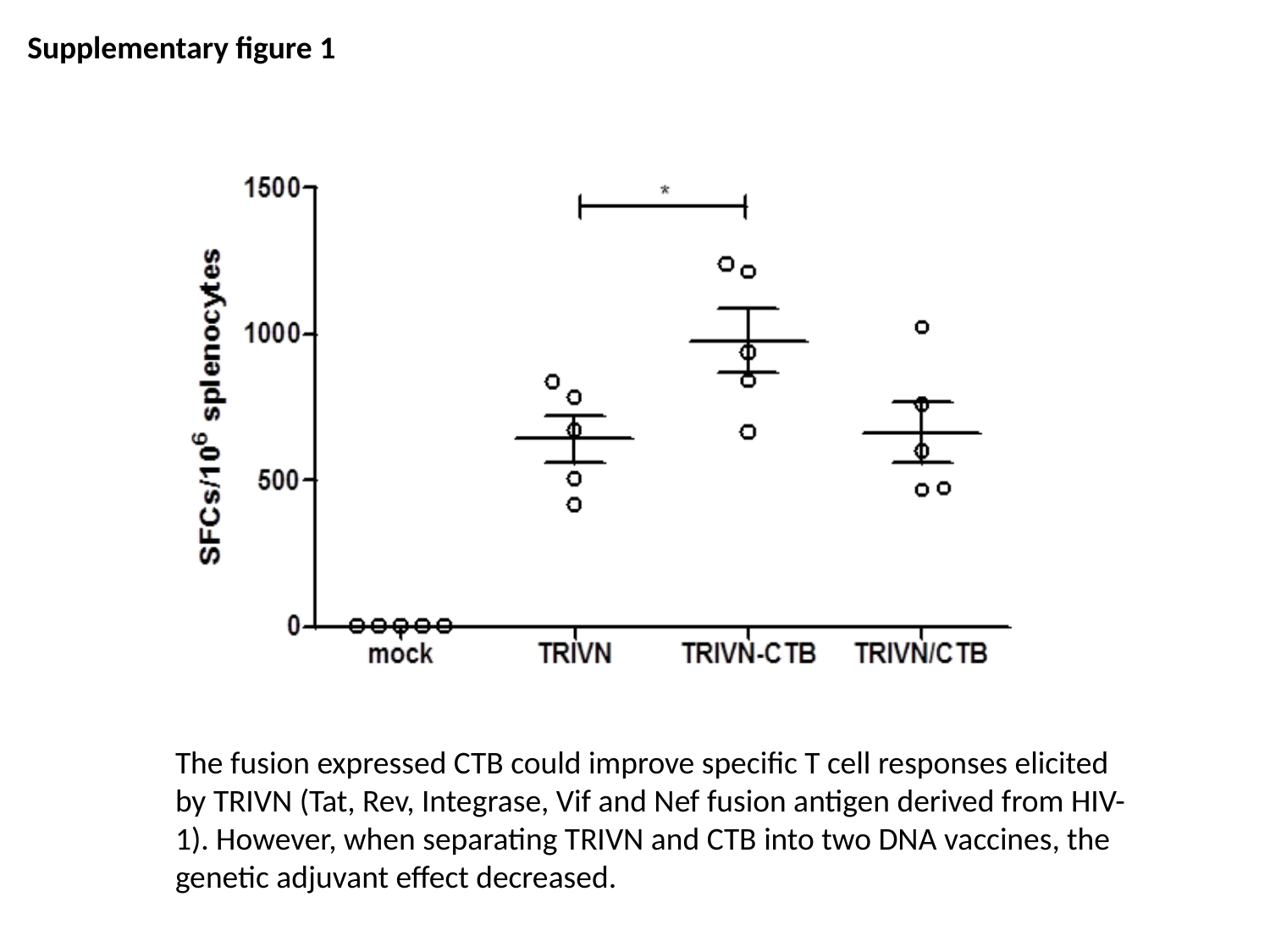

Supplementary figure 1
The fusion expressed CTB could improve specific T cell responses elicited by TRIVN (Tat, Rev, Integrase, Vif and Nef fusion antigen derived from HIV-1). However, when separating TRIVN and CTB into two DNA vaccines, the genetic adjuvant effect decreased.
